# Surveillance of Antibiotic Resistance among Hospital- and Community-Acquired Toxigenic *Clostridium difficile* Isolates over 5-Year Period in Kuwait

**DOI:** 10.1371/journal.pone.0161411

**Published:** 2016-08-18

**Authors:** Wafaa Y. Jamal, Vincent O. Rotimi

**Affiliations:** Anaerobe Reference Laboratory and Microbiology Department, Faculty of Medicine, Kuwait University, Jabriya, Kuwait; Leibniz-Institute DSMZ, GERMANY

## Abstract

*Clostridium difficile* infection (CDI) is a leading and an important cause of diarrhea in a healthcare setting especially in industrialized countries. Community-associated CDI appears to add to the burden on healthcare setting problems. The aim of the study was to investigate the antimicrobial resistance of healthcare-associated and community-acquired *C*. *difficile* infection over 5 years (2008–2012) in Kuwait. A total of 111 hospital-acquired (HA-CD) and 35 community-acquired *Clostridium difficile* (CA-CD) clinical isolates from stool of patients with diarrhoea were studied. Antimicrobial susceptibility testing of 15 antimicrobial agents against these pathogens was performed using E test method. There was no evidence of resistance to amoxicillin-clavulanic acid, daptomycin, linezolid, piperacillin-tazobactam, teicoplanin and vancomycin by both HA-CD and CA-CD isolates. Metronidazole had excellent activity against CA-CD but there was a 2.9% resistance rate against HA-CD isolates. Ampicillin, clindamycin, levofloxacin and imipenem resistance rates among the HC-CD vs. CA-CD isolates were 100 vs. 47.4%; 43 vs. 47.4%; 100 vs. 100% and 100 vs. 89%, respectively. An unexpected high rifampicin resistance rate of 15.7% emerged amongst the HA-CD isolates. In conclusion, vancomycin resistance amongst the HA-CD and CA-CD isolates was not encountered in this series but few metronidazole resistant hospital isolates were isolated. High resistance rates of ampicillin, clindamycin, levofloxacin, and imipenem resistance were evident among both CA-CD and HA-CD isolates. Rifampicin resistance is emerging among the HA-CD isolates.

## Introduction

*Clostridium difficile* is a Gram-positive, spore-forming anaerobic bacteria. It has an important role in hospital-acquired diarrhoea ranging from mild cases to severe pseudomemraneous colitis, collectively named *C*. *difficile* infection (CDI). The number of CDI has increased dramatically in term of frequency, severity, occurrence in outbreaks setting and recurrence rate since 2002 in North America [1], Europe [2] and Australia [3]. This is partially related to the emergence of the hypervirulent strains i.e. *C*. *difficile* B1/ NAP1/027 [4]. Another hypervirulent strain i.e. *C*. *difficile* ribotype 078 has emerged, whose incidence increased from 3 to 13% during 2005–2008 in the Netherlands [4]. In addition, new risk groups have been added to the list which include community-onset CDI [5, 6], CDI in children as well as peripartum ladies [7].

Antimicrobial therapy plays an important role in the development of CDI. This risk increases if *C*. *difficile* is resistant to the offending or the used antimicrobial agent [8]. One of the main theories for the rise in the reported cases as well as outbreaks of CDI is the circulation of flouroquinolone-resistant *C*. *difficile* ribotype 027 at the same time as the wild use of flouroquinolones in hospitals [9]. Antibiotic resistance are important in the emergence of epidemic clones and persistence of specific types over time in the hospitals.

CDI plays a significant burden on the healthcare setting and financial resources. Treatment of *C*. *difficile* is difficult because of its direct causal relationship with antibiotic use. The two most commonly prescribed antimicrobial agents for treatment of CDI are metronidazole and vancomycin with high recurrence rates. Antimicrobial susceptibility testing is not done routinely for anaerobes including *C*. *difficile*. Although *C*. *difficile* has been reported as susceptible to metronidazole and vancomycin, there are some reports of reduced susceptibility of *C*. *difficile* to metronidazole [10,11]. High percentage of multi-drug resistant (resistant to 3 or more drugs) *C*. *difficile* was found in certain European hospitals [12]. There is no clear relationship or association between treatment failure and reduced susceptibility or resistance to metronidazole or vancomycin. The aim of this study was to investigate the trend of antibiotic resistance of healthcare-associated and community-acquired *C*. *difficile* infection (HA-CDI; CA-CDI) over 5 years (2008–2012) in Kuwait.

## Methods

### Definitions

Diarrhea was defined as loose stools, i.e. taking the shape of the container or corresponding to Bristol stool chart types 5–7, plus a stool frequency of three stools in 24 h or fewer consecutive hours or more frequently than is normal for the individual (definition of World Health Organization, http://www.who.int/topics/diarrhoea) [13, 14, 15]. According to the European Centre for Disease Prevention and Control [16], an episode of CDI was defined as a patient with diarrhea whose stool takes the shape of the container, and it is positive for *C*. *difficile* toxin A and/or B without other etiology or endoscopic evidence of pseudomembranous colitis.

CA-CDI was defined, in this study, as the onset of symptoms occurring while the patient was outside a healthcare facility and the patient had not been discharged from a healthcare facility within 12 weeks before symptom onset (community onset/community-acquired); or the onset of symptoms occurring within 48 h upon admission to a healthcare facility and the patient had no prior stay in a healthcare facility within the 12 weeks prior to symptom onset (healthcare facility onset; community-acquired) [16].

### Bacterial strains

Stool samples were collected from diarrheagenic patients suspected of *C*. *difficile* infection acquired in the hospital or community from 2008–2012. They were processed in the Anaerobe Reference Laboratory, (ARL, Ministry of Health), Faculty of Medicine, Kuwait. Stool specimens were cultured on selective media (cycloserine-cefoxitin fructose agar; CCFA, Oxoid limited, Basingstoke, Hampshire, UK) after heat-shock procedure. Suspected *C*. *difficile* isolates were identified by Gram staining, typical morphology on agar plates, as well as characteristic odour. Then it was identified further by biochemical tests using API 20A (bioMerieux, Marcy I’Etoile, France) and confirmed by VITEK MS (bioMerieux). Toxin production was investigated by GeneXpert^TM^ C difficile assay (Cepheid, Sunnyvale, CA, USA) and EIA method using C. diff Quik Chek Complete kit (QCC) (TechLab, Blacksburg, VA, USA) according to manufacturer’s instructions.

### Antimicrobial susceptibility testing (AST)

AST was performed for the following antimicrobial agents: ampicillin, amoxicillin-clavulanic acid, clindamycin, daptomycin, erythromycin, imipenem, levofloxacin, linezolid, meropenem, metronidazole, piperacillin-tazobactam, rifampicin, teicoplanin, tigecycline and vancomycin. AST was done by determining the minimum inhibitory concentrations (MICs) of the above antibiotics using the E test method (bioMerieux), according to the manufacturer’s instructions, on Brucella agar supplemented with hemin (5μg/ml); Sigma-Aldrich, St. Louis, Missouri, USA), Vitamin K_1_ (1μg/ml; HiMedia Laboratories Pvt. Ltd, Mumbai, India) and laked sheep blood (5% v/v). Resistance profiles of the isolates were determined according to the interpretative criteria recommended by the CLSI [17] for ampicillin, amoxicillin-clavulanic acid, piperacillin-tazobactam, imipenem, meropenem, clindamycin and metronidazole, while EUCAST, 2014 were applied for levofloxacin, vancomycin, tigecycline, daptomycin, and rifampicin (www.eucast.org/clinical_breakpoints). If no standard MIC breakpoint had been defined such as for linezolid, erythromycin, and teicoplanin, susceptibility breakpoint was considered to be ≤4 μg/ml [18], ≤8μg/ml [19], and ≤8μg/ml (*Staphylococcus aureus* according to CLSI), respectively [17], were used. *C*. *difficile* ATCC 700057 and *Eubacterium lentum* ATCC 43055 were used as quality control organisms with each run. MIC_50_ and MIC_90_ values for each antibiotic was calculated by using Microsoft Excel.

### PCR ribotyping

PCR ribotyping of all isolates was performed as previously described [20].

### Statistical analysis

Fisher’s exact test (two-sided) was used to test for difference between CA-CD and HA-CD with those differences showing p <0.05 deemed to be statistically significant.

### Ethics statement

Collection of the strains was conducted according to the Declaration of Helsinki and with particular institutional ethical and professional standards. A written informed consent was not obtained from patients or parents of children because the bacterial isolates studied were collected from the routine work of clinical microbiology laboratory for patient care and no additional clinical specimens were collected for the purpose of the study. It is a standard practice not to get written informed consent for use of bacterial isolates unlinked to patient identity from the routine clinical laboratory. Therefore, the waiver for informed consent was granted and the study was approved by the Medical Ethics Committee of Ministry of Health, Kuwait (permit number 2093/MTT).

## Results

A total of 111 hospital-acquired and 35 community-acquired toxigenic *C*. *difficile* isolates were analyzed. The distribution of HA-CD and CA-CD over 5 years is shown in Table 1.

**Table 1:** Number of hospital-acquired *C*. *difficile* (HA-CD) and community-acquired *C*. *difficile* (CA-CD) isolates per year.

	2008	2009	2010	2011	2012	Total
CA-CD	5	8	7	8	7	35
HA-CD	17	31	13	43	7	111

HA-CD = Hospital-acquired *Clostridium difficile* isolates; CA-CD = community-acquired *C*. *difficile* isolates.

Amoxicillin-clavulanic acid (MIC_90_ = 1 vs. 0.75 μg/ml), daptomycin (MIC_90_ = 4 vs. 0.75 μg/ml), linezolid (MIC_90_ = 3 vs. 1 μg/ml), metronidazole (MIC_90_ = 0.75 vs. 0.125 μg/ml), piperacillin-tazobactam (MIC_90_ = 6 vs. 6 μg/ml), teicoplanin (MIC_90_ = 0.5 vs. 0.38 μg/ml) and vancomycin (MIC_90_ = 3 vs. 1.5 μg/ml) had excellent activities against both HA-CD and CA-CD isolates, respectively as shown in Table 2.

**Table 2:** Antimicrobial susceptibility results of 111 hospital-acquired *C*. *difficile* (HA-CD) and 35 community-acquired *C*. *difficile* (CA-CD) isolates.

Antibiotic (breakpoints in μg/ml)	MIC_50_ (in μg/ml)		MIC_90_ (in μg/ml)		% of resistance		*P* value
	CA-CD	HA-CD	CA-CD	HA-CD	CA-CD	HA-CD	
Ampicillin (0.5)	1	0.5	2	1.5	100	47.4	<0.001
Amoxicillin-clavulanic acid (4/2)	0.25	0.25	0.75	1	0	0	-
Clindamycin (2)	2	0.2	3	>256	43	47.4	0.70
Daptomycin (4)	0.38	0.38	0.75	4	0	0	-
Erythromycin (8)	0.25	256	0.38	256	14.3	58	<0.001
Imipenem (4)	>32	32	>32	>32	100	89	0.07
Levofloxacin (4)	>32	32	>32	32	100	100	-
Linezolid (4)	0.75	0.75	1	3	0	0	-
Meropenem (4)	2	0.75	>32	1	43	0	<0.001
Metronidazole (8)	0.094	0.125	0.125	0.75	0	2.9	1.00
Piperacillin-tazobactam (32/4)	4	0.19	6	6	0	0	-
Rifampicin (0.004)	<0.002	0.001	<0.002	32	0	15.7	0.014
Teicoplanin (8)	0.25	0.25	0.38	0.5	0	0	-
Tigecycline (0.25)	0.023	0.016	0.032	0.25	0	5.3	0.34
Vancomycin (4)	1	1	1.5	3	0	0	-

HA-CD = Hospital-acquired *Clostridium difficile* isolates; CA-CD = community-acquired *C*. *difficile* isolates

There was no significant difference in the resistance against HA-CD and CA-CD isolates for the above antimicrobial agents. All community isolates were resistant to ampicillin compared to only 4.7.4% of the hospital isolates (p<0.001); (MIC_90_ = 2 vs. 1.5 μg/ml). The HA-CD isolates were more resistant to clindamycin than the CA-CD (MIC_90_ ≥256 vs. 3 μg/ml) with resistance slightly higher among HA-CD (47.4% vs. 43%; p = 0.70). Similarly, resistance to erythromycin was more common among HA-CD compared to CA-CD (MIC_90_ = 256 vs. 0.38 μg/ml and 58% vs. 14.3%; p<0.001). Resistance to imipenem was very common among both HA-CD and CA-CD isolates MIC_90_ >32 vs. >32 μg/ml and 89% and 100%; p = 0.07; while resistance to meropenem was more common among CA-CD compared to HA-CD (MIC_90_ = >32 vs. 1 μg/ml; 43 vs. 0%; p <0.001). Rifampicin (MIC_90_ = <0.002 vs. 32 μg/ml) resistance was seen commonly among the hospital isolates compared to the community isolates (15.7 vs. 0%; p = 0.014). Tigecycline resistance was seen more often among the hospital isolates compared to the community isolates (5.3 vs. 0%) although the difference is not statistically significant (p = 0.34).

Table 3 shows the PCR ribotypes for both CA-CD and HA-CD isolates. Among the CA-CDI isolates, eleven (31.4%) isolates belonged to PCR ribotype 139, 7 (20%) isolates belonged to ribotype 097, 6 (17.1%) isolates belonged to ribotype 070. Four (11.4%) each of the 2 ribotypes belonged to ribotype 056 and 179, and only 3 (8.6%) isolates belonged to ribotype 014.

**Table 3:** *C*. *difficile* PCR ribotypes of CA-CDI and HA-CDI.

PCR Ribotype	CA-CDI		HA-CDI	
	Number	Percentage	Number	Percentage
**001**	**-**	**-**	**21**	**18.9**
**002**	**-**	**-**	**23**	**20**
**003**	**-**	**-**	**12**	**10.8**
**005**	**-**	**-**	**4**	**3.6**
**014**	**3**	**8.6**	**6**	**5.4**
**029**	**-**	**-**	**4**	**3.6**
**056**	**4**	**11.4**	**3**	**2.7**
**057**	**-**	**-**	**5**	**4.5**
**070**	**6**	**17.1**	**-**	**-**
**083**	**-**	**-**	**3**	**2.7**
**097**	**7**	**20**	**-**	**-**
**107**	**-**	**-**	**3**	**2.7**
**126**	**-**	**-**	**14**	**12.6**
**139**	**11**	**31.4**	**-**	**-**
**159**	**-**	**-**	**4**	**3.6**
**177**	**-**	**-**	**3**	**2.7**
**179**	**4**	**11.4**	**-**	**-**
**195**	**-**	**-**	**6**	**5.4**
**Total no**	**35**		**111**	

As shown in Table 3, 14 distinct, genotypically different ribotypes were identified among the 111 HA-CD isolates. The commonest PCR ribotypes were the following: 23 (20%) isolates belonged to ribotype 002, 21 (18.9%) ribotype 001, 14 (12.6%) ribotype 126, and 12 (10.8%) ribotype 003. The remaining isolates belonged to 10 different ribotypes which were 014 (6, 5.4%), 195 (6, 5.4%), 057 (5, 4.5%), 4 (3.6%) each of ribotype 005, 029, and 159, 3 (2.7%) each of ribotype 056, 083, 107 and 177.

The MIC_50_, MIC_90_ and percentage of resistance to the common PCR ribotypes in both CA-CDI and HA-CDI are shown in Table 4. Only PCR ribotypes 014 and 056 were common to both CA-CDI and HA-CDI. Resistance to erythromycin, clindamycin and rifampicin was more common among HA-CD ribotypes compared to CA-CD (66.6, 78, 28.6% vs. 35, 7.1 and 0%, respectively). However, the resistance to amoxicillin and meropenem was more common among the CA-CD ribotypes compared to HA-CD (100, 48.9% vs. 55, and 0%, respectively).

**Table 4:** Susceptibility and summary of MIC data of different antimicrobial agents and percentage of resistance antibiotics against *C*. *difficile* isolated from CA-CDI and HA-CDI, by PCR ribotypes.

PCR ribotype	Antibiotics	CA-CDI			HA-CDI		
		MIC_50_	MIC_90_	% of resistance	MIC_50_	MIC_90_	% of resistance
RT 014	Ampicillin	0.75	2	100	0.5	1.0	50
	Amox-clav	0.125	0.5	0	0.25	1.0	0
	Clindamycin	1.0	4	35	0.75	128	66.6
	Erythromycin	0.19	0.50	7.1	192	256	78
	Levofloxacin	32	32	100	24	32	100
	Meropenem	1.0	24	48.9	0.5	0.75	0
	Metronidazole	0.064	0.19	0	0.094	0.19	0
	Rifampicin	0.003	0.003	0	0.002	2	28.6
	Vancomycin	0.75	1.0	0	1	2	0
RT 056	Ampicillin	1	4	100	0.25	0.75	66.6
	Amox-clav	0.25	1.0	0	0.5	1.0	0
	Clindamycin	1	3	50	0.38	>256	66.6
	Erythromycin	0.5	8	25	32	256	100
	Levofloxacin	32	32	100	32	32	100
	Meropenem	2	8	25	0.75	1	0
	Metronidazole	0.032	0.5	0	0.064	0.75	0
	Rifampicin	0.002	0.002	0	0.002	0.012	33.3
	Vancomycin	0.25	0.75	0	0.38	0.75	0
RT 070	Ampicillin	1.0	6	100	-	-	-
	Amox-clav	0.047	1.0	0	-	-	-
	Clindamycin	0.5	2	16.7	-	-	-
	Erythromycin	2	24	33.3	-	-	-
	Levofloxacin	12	>32	100	-	-	-
	Meropenem	0.125	16	33.3	-	-	-
	Metronidazole	0.047	0.094	0	-	-	-
	Rifampicin	0.002	0.002	0	-	-	-
	Vancomycin	0.016	0.094	0	-	-	-
RT 097	Ampicillin	8	12.8	100	-	-	-
	Amox-clav	0.25	0.75	0	-	-	-
	Clindamycin	1.0	2	14.3	-	-	-
	Erythromycin	0.5	8	4.3	-	-	-
	Levofloxacin	32	>32	100	-	-	-
	Meropenem	1	4	28.6	-	-	-
	Metronidazole	0.38	1.0	0	-	-	-
	Rifampicin	0.002	0.004	0	-	-	-
	Vancomycin	0.75	1.5	0	-	-	-
RT 139	Ampicillin	1.0	2	100	-	-	-
	Amox-clav	0.5	0.75	0	-	-	-
	Clindamycin	0.5	16	45.5	-	-	-
	Erythromycin	0.25	4	9	-	-	-
	Levofloxacin	32	32	100	-	-	-
	Meropenem	2	24	27.3	-	-	-
	Metronidazole	0.19	0.38	0	-	-	-
	Rifampicin	0.002	0.002	0	-	-	-
	Vancomycin	0.5	1.0	0	-	-	-
RT 179	Ampicillin	1	2	100	-	-	-
	Amox-clav	0.19	0.25	0	-	-	-
	Clindamycin	0.5	3	25	-	-	-
	Erythromycin	0.5	1.0	0	-	-	-
	Levofloxacin	32	32	100	-	-	-
	Meropenem	1	32	25	-	-	-
	Metronidazole	0.064	0.125	0	-	-	-
	Rifampicin	<0.002	0.002	0	-	-	-
	Vancomycin	0.125	0.5	0	-	-	-
RT 001	Ampicillin	-	-	-	1.0	4	71.4
	Amox-clav	-	-	-	0.25	1.0	0
	Clindamycin	-	-	-	1.5	128	23
	Erythromycin	-	-	-	16	256	62
	Levofloxacin	-	-	-	32	32	100
	Meropenem	-	-	-	0.5	1.0	0
	Metronidazole	-	-	-	0.125	0.5	0
	Rifampicin	-	-	-	0.004	1.5	23.8
	Vancomycin	-	-	-	1.0	1.5	0
RT 002	Ampicillin	-	-	-	1.0	1.0	78.3
	Amox-clav	-	-	-	0.19	1.0	0
	Clindamycin	-	-	-	2	256	56.5
	Erythromycin	-	-	-	256	256	60.8
	Levofloxacin	-	-	-	32	32	100
	Meropenem	-	-	-	0.5	1	0
	Metronidazole	-	-	-	0.19	0.5	0
	Rifampicin	-	-	-	0.002	32	17.4
	Vancomycin	-	-	-	0.75	2	0
RT 003	Ampicillin	-	-	-	0.25	1.5	41.7
	Amox-clav	-	-	-	0.125	1	0
	Clindamycin	-	-	-	96	256	41.7
	Erythromycin	-	-	-	64	256	58.3
	Levofloxacin	-	-	-	32	32	100
	Meropenem	-	-	-	0.38	1	0
	Metronidazole	-	-	-	0.094	0.5	0
	Rifampicin	-	-	-	<0.002	0.002	8.3
	Vancomycin	-	-	-	0.75	1.5	0
RT 126	Ampicillin	-	-	-	0.25	1.5	21.4
	Amox-clav	-	-	-	0.38	1	0
	Clindamycin	-	-	-	2	256	42.9
	Erythromycin	-	-	-	48	256	50
	Levofloxacin	-	-	-	32	32	100
	Meropenem	-	-	-	0.5	1.0	0
	Metronidazole	-	-	-	0.094	0.5	0
	Rifampicin	-	-	-	0.002	0.002	0
	Vancomycin	-	-	-	0.5	2	0

RT = PCR ribotype

## Discussion

Knowing the antimicrobial susceptibility is critically important when treating patients with CDI in hospital as well as in community settings. Only one study on hospital-acquired *C*. *difficile* antimicrobial susceptibility in Kuwait has been published and that was 13 years ago [21]. In the previous study, antimicrobial susceptibility of 15 antimicrobial agents was determined by E test methodology for hospital acquired *C*. *difficile* isolates collected from patients in intensive care units of 3 hospitals over a period of 2 years in Kuwait [21]. There have been no study in Kuwait or elsewhere comparing the susceptibility of HA-CD and CA-CD.

In this study, we have evaluated 15 antimicrobial agents, including the two antibiotics currently used as standard therapy for CDI, vancomycin and metronidazole, against 111 hospital-acquired and 35 community-acquired *C*. *difficile* isolates. Our data showed that 2.9% of HA-CD were resistant to metronidazole while all CA-CD isolates were susceptible. However, worthy of note was the higher MIC_90_ values demonstrated by current CA-CD isolates compared to earlier study for HA-CD, 0.75 vs. 0.19μg/ml, respectively. This is in contrast to the surveillance studies in Spain and Texas, which reported much higher resistance rates of clinical *C*. *difficile* isolates to metronidazole (6.3% and 13.3%), respectively [22, 23], unlike lower resistance level of 0.11% reported in the European surveillance study by Freeman *et al* [24]. In the Pelaez *et al* study, metronidazole resistance was heterogeneous and not related to the presence of *nim* genes [22]. All our CA-CD and HA-CD isolates were susceptible to vancomycin howbeit with higher MIC_90_ values compared to earlier study for HA-CD (3.0 vs. 0.75μg/ml). This finding is consistent with previous reports [25, 26] which demonstrated MIC_90_ of 0.75 and 2μg/ml, respectively. Contrastingly, a pan-European surveillance study in 22 European countries reported a 0.9% vancomycin resistance rate [24]. However the role of vancomycin resistant *C*. *difficile* is not clear because of the high concentration of vancomycin in the colon of patients after oral vancomycin therapy [27].

In our study, non-susceptibility to imipenem was high in both CA-CD and HA-CD (100 vs. 89%) whereas meropenem resistance was high for only CA-CD (43 vs. 0%). Imipenem resistance in this study is similar to our previous study [21] for HA-CD isolates as well as to the study in Poland [25] which reported that 86%, 87.9%, respectively, of their strains were resistant to imipenem. These reports differ from other studies in Korea and Europe where resistance of HA-CD isolates to imipenem was relatively low 7.4% and 8%, respectively [26, 28]. While resistance to meropenem was zero in the previous study in Kuwait as well as in Australia [21, 26] for HA-CD isolates, it was relatively high with the CA-CD isolates. Imipenem and meropenem have been used liberally in Kuwait hospitals for the last two decades, which may have led to the selection pressure for resistance to imipenem and meropenem secondary to drug exposure.

Clindamycin resistance was observed in 43% and 47% of CA-CD and HA-CD isolates, respectively, a finding similar to results of our previous study (48%) [21] and a European study (49.62%) [24] but much higher than that reported previously in Poland (27.7%) [25]. It is, however, lower than reports from Australia [26] and Korea [28] where 84.3% and 81%, respectively were resistant. Interestingly, there was a remarkable difference in the resistance levels to erythromycin which was observed in 14.3% of CA-CD isolates compared to 58% of HA-CD isolates. The Korean [28] and Polish [25] studies reported high level resistance to erythromycin, of 80% and 85.5%, respectively.

Rifampicin resistance was 16% among our HA-CD isolates. This is similar to reports from Poland and Europe of 13.40% and 18%, respectively [24, 25], but unlike reports from Australia were the resistance rate to rifaximin was almost 0% [26]. The relatively high rifampicin resistance rate in our study may be explained, in part, by the concurrent high rifampicin resistance rates of 10% amongst the *Staphylococcus aureus* isolates in Kuwait [29] and of 9.2% rate amongst the *Mycobacterium tuberculosis* isolates [30]. In Poland, rifampicin resistance has emerged in an outbreak of *C*. *difficile* ribotype 046 in patients on long term treatment of rifampicin for tuberculosis [31].

We found a striking difference between the susceptibilities of our isolates (HA-CD and CA-CD) to the quinolones, in particular levofloxacin, and the Australian isolates. While all our isolates were resistant as in our previous study of 97% to trovafloxacin, only 3.4% of the Australian isolates were reported as resistant to moxifloxacin [26].

One of the limitations of this study is the relatively small number of CA-CDI isolates, which is probably a true reflection of the paucity of CDI in our country. The second limitation was the use of E test for testing the susceptibility of the isolates to metronidazole. Although CLSI recommends agar dilution method to test for metronidazole MIC, comparison of MICs determined by agar dilution and E test has shown good correlation (+/- 2 dilutions) of 86.6, 95.9, and 99% for metronidazole, vancomycin and teicoplanin, respectively, as reported by Barbut *et al* [32]. In addition, antimicrobial susceptibility testing of *C*. *difficile* by E test method has been well documented in previous reports in the literature [21, 24, 31].

## Conclusions

We did not encounter any vancomycin-resistant isolate amongst the HA-CD and CA-CD isolates in this series but few metronidazole resistant hospital isolates were isolated. Ampicillin, clindamycin, levofloxacin and imipenem resistance were evidently at unacceptable levels for both CA-CD and HA-CD isolates. We noted that rifampicin resistance is emerging among the HA-CD isolates and this calls for caution in the use of rifampicin for infections other that *Mycobacterium tuberculosis*. Therefore, we recommend periodic surveillance and regular antimicrobial susceptibility testing for all toxigenic *C*. *difficile* isolates as an informed guide to empiric antibiotic use.
